# Genicular Artery Embolization for Chronic Knee Pain: Expert Consensus Recommendations on Indications, Technique and Clinical Care Using a Delphi Process

**DOI:** 10.1007/s00270-026-04547-8

**Published:** 2026-07-22

**Authors:** A. Taheri Amin, J. Golzarian, K. Abd El Tawab, L. Abu-Gharbieh, O. Ahmed, A. Assis, S. A. Astani, CA. Binkert, Francisco Cesar Carnevale, F. Cavalheiro, F. Collettini, M. P. Correa, A. Dablan, K. Damodharan, Y. Epelboym, A. M. Fernández, D. Filippiadis, G. S. Goh, A. Guermazi, Z. Haskal, A. M. Ierardi, M. Katoh, M. Little, Y. Okuno, S. Padia, N. Rostambeigi, M. Sapoval, B. Taslakian, R. Uberoi, H. Vieweg, F. Ziayee, P. Minko

**Affiliations:** 1 Department of Diagnostic and Interventional Radiology, Charité Universitätsmedizin Berlin, Corporate Member of Freie Universität Berlin and Humboldt-Universität Zu Berlin, Berlin, Germany; 2 Department of Diagnostic and Interventional Radiology, Medical Faculty, University Hospital Duesseldorf, 40225 Duesseldorf, Germany; 3 Department of Radiology, University of Minnesota, Minneapolis, MN USA; 4 North Star Vascular Interventional Institute, Minneapolis, MN USA; 5 Department of Radiology, Interventional Radiology Unit, Ain Shams University Hospitals, Abbasia, Cairo, Egypt; 6 Joint and Vascular Institute, Libertyville, IL USA; 7 Department of Radiology, University of Utah, Salt Lake City, IL USA; 8 Interventional Radiology and Endovascular Surgery Department, University of Sao Paulo Medical School, Dr. Enéas de Carvalho Aguiar Avenue, São Paulo, Cerqueira César 25505403000 Brazil; 9 University of Minnesota, 420 Delaware St.SE, Mayo B228, RadiologyMinneapolis, MN 55455 USA; 10 Imagiology Department, Gaia Espinho Local Health Unit, V. N. de Gaia, Portugal; 11 Imagiology Department, CUF Porto Hospital, Porto, Portugal; 12 BIH Charité Clinician Scientist Program, BIH Biomedical Innovation Academy, Berlin Institute of Health, Charité-Universitätsmedizin Berlin, 10117 Berlin, Germany; 13 Instituto Vascular de Passo Fundo (Invasc), Passo Fundo, RS Brazil; 14 School of Medicine, Universidade de Passo Fundo, Passo Fundo, RS Brazil; 15 School of Medicine, Atitus Educação, Passo Fundo, RS Brazil; 16 Department of Radiology, Basaksehir Cam and Sakura City Hospital, Istanbul, Turkey; 17 Department of Vascular and Interventional Radiology, Madras Institute of Orthopedics and Traumatology (MIOT) Hospitals, Chennai, India; 18 Department of Radiology, Brigham and Women’s Hospital, Boston, MA USA; 19 Vascular and Interventional Radiology Department, University Hospital of León, Calle Altos de Nava, 24080 León, SN Spain; 20 Associate Professor of Diagnostic and Interventional Radiology, 2nd Department of Radiology, Medical School, University General Hospital “ATTIKON”, National and Kapodistrian University of Athens, Athens, Greece; 21 Department of Radiology, Alfred Health, Melbourne, VIC Australia; 22 Department of Surgery, School of Translational Medicine, Monash University, Victoria, Australia; 23 Quantitative Imaging Center, Department of Radiology, Boston University School of Medicine, 820 Harrison Avenue, FGH Building, 4Th Floor, Boston, MA 02118 USA; 24 Department of Radiology, VA Boston Healthcare System, 1400 VFW Parkway, , West Roxbury, MA 02132 USA; 25 Department of Radiology and Medical Imaging/Interventional Radiology. Professor of Radiology, University of Virginia School of Medicine, Charlottesville, USA; 26 Department of Diagnostic and Interventional Radiology, Foundation IRCCS Ca’ Granda Ospedale Maggiore Policlinico, 20122 Milan, Italy; 27 Department of Oncology and Hemato-Oncology, University of Milan, 20122 Milan, Italy; 28 Department of Diagnostic and Interventional Radiology, Helios Klinikum Krefeld, Lutherplatz, Krefeld, Germany; 29 University Department of Radiology, Royal Berkshire NHS Foundation Trust, Reading, UK; 30 University of Reading, Reading, UK; 31 Department of Interventional Radiology, Okuno Clinic Tokyo, Tokyo, Japan; 32 Department of Radiology, David Geffen School of Medicine, at the University of California Los Angels, Los Angeles, CA USA; 33 Vascular Interventional Radiology, Mallinckrodt Institute of Radiology, Washington University School of Medicine, St. Louis, MO 63110 USA; 34 Department of Vascular and Oncological Interventional Radiology, Hopital Europe´en Georges Pompidou, APHP, Paris, France; 35 Department of Radiology, NYU Langone Health, New York, USA; 36 Department of Radiology, John Radcliffe Hospital, Oxford University Hospitals NHS Trust, Headington, Oxford, OX3 9DU UK; 37 Faculty, Department of Radiology and Neuroradiology, Asklepios Klinik Nord, Academic Teaching Hospital of the University of Hamburg, Hamburg, Germany

**Keywords:** Genicular artery embolization, Knee osteoarthritis, Chronic knee pain, Delphi consensus

## Abstract

**Purpose:**

To develop expert-consensus recommendations for patient selection, technique and clinical management in genicular artery embolization (GAE) using a Delphi process.

**Materials and Methods:**

A working group developed a 75-statement questionnaire. A panel of musculoskeletal and interventional radiologists (IRs), selected based on clinical experience, scientific expertise and geographic diversity, scored each response using a 10-point Likert-scale across three rounds. Consensus was predefined as ≥ 75% of ratings ≥ 7/10.

**Results:**

Twenty-nine IRs completed all three rounds. Consensus inclusion criteria for GAE include knee pain refractory to conservative treatment for ≥ 3 months due to osteoarthritis, tendinopathies or prior knee surgery and recurrent hemarthrosis (median 9[IQR 7–10]; 86% ≥ 7). Pre-procedural assessment should include standardized outcome measures, clinical examination and knee radiographs. Contrast-enhanced MRI is optional for osteoarthritis phenotyping and grading of synovitis, differential diagnosis and outcome prediction (8[7–10]; 79% ≥ 7). Via an ipsilateral antegrade transfemoral access, all visible genicular arteries should be catheterized and embolized upon detection of a hypervascular blush (9[7–10]; 76% ≥ 7). No evidence of superiority of either temporary or permanent embolic agents in terms of safety or efficacy has been demonstrated (10[8–10]; 86% ≥ 7). Structured long-term follow-up is recommended, with clinical success defined as achievement of the minimal clinically important difference or individual patient satisfaction (9[7–10]; 83% ≥ 7). Contralateral or repeat GAE may be considered for bilateral knee pain, insufficient response or pain recurrence (8[7–10]; 76% ≥ 7).

**Conclusion:**

This Delphi study establishes expert-derived consensus recommendations for GAE, emphasizing broad indications, patient-tailored technique and an active role of the IR in multidisciplinary longitudinal care.

**Graphic Abstract:**

**Figure:**
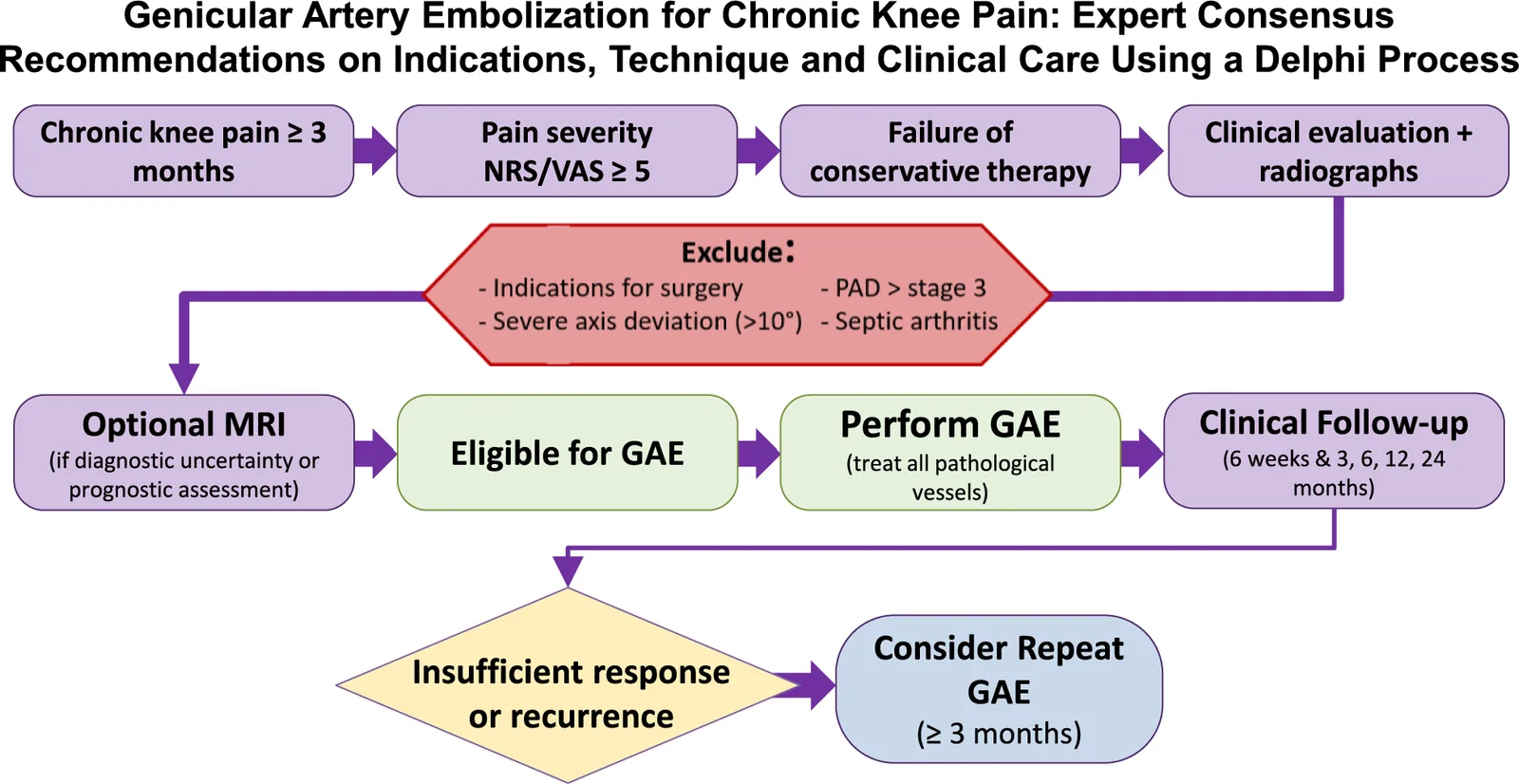

**Supplementary Information:**

The online version contains supplementary material available at https://doi.org/10.1007/s00270-026-04547-8.

## Introduction

Genicular artery embolization (GAE) is emerging as a minimally invasive treatment option for patients with chronic knee pain refractory to conservative treatment. GAE has demonstrated high technical success rates with clinically meaningful improvements sustained up to 4 years [1–7]. As adoption of GAE increases, heterogeneity in patient selection, procedural technique and clinical management have become apparent [8]. Most notably, management of patients with severe osteoarthritis, number of arteries to be embolized and choice between temporary and permanent embolic agents, including their differing embolization endpoints, remain the most debated aspect critically influencing clinical outcome [9–12]. While position statements address individual aspects of these debates, comprehensive, consensus-based recommendations are still lacking [13, 14].

To address this knowledge gap, a Delphi study was conducted among an international panel of IRs with expertise in GAE. The resulting consensus recommendations on patient selection, technique and clinical care are intended to guide daily practice, harmonize quality standards across centers and provide a framework for high-quality evidence to support the integration of GAE into clinical guidelines.

## Materials and Methods

Two IRs with 33 and 18 years of experience (J.G.; P.M.) and a diagnostic musculoskeletal radiologist (A.G.) defined 72 open-ended questions addressing patient selection, procedural technique and clinical care during a joint session (Supplementary Table 1). Based on the number of GAE procedures performed (≥ 50), published research (≥ 3 peer-reviewed publications on embolotherapy within the past five years) and geographic diversity, the working group invited 31 IRs, of whom 29 agreed to participate (two did not respond). Panelists remained unaware of the identity of co-panelists.

The open-ended questionnaire was digitized by the study team (A.T.A.; L.A.) and distributed via an online survey platform (SurveyMars). Responses to the open-ended round were anonymized by the study team and presented to the working group (J.G.; P.M.; A.G.), which consolidated overlapping responses, excluded redundant or out-of-scope items and refined the remaining responses into 75 statements. These statements were digitized and distributed via the same platform. Panelists rated each statement on a 10-point Likert-scale (0 = strongly disagree, 10 = fully agree) during the second round. Statements not reaching consensus were resubmitted in a third round, accompanied by the percentage agreement from the preceding round. Panelists were allowed to revise their ratings in light of group opinion. Between rounds, responses were analyzed and anonymized exclusively by the study team. The panelists and the working group were unaware of which panelist had provided which response. Consensus was predefined as ≥ 75% of ratings ≥ 7 [15, 16]. Statements failing to meet consensus in both rounds were classified as not amenable to consensus. For each item, number of responses, median with interquartile range (IQR) and percentage ≥ 7 were calculated. Round-to-round stability of ratings was assessed using the Wilcoxon signed-rank test, and inter-rater agreement was quantified using Kendall's coefficient of concordance (W). Statistical analyses were performed using Microsoft Excel (Excel 2024) and R (Version 4.4.1).

## Results

**Fig. 1:**
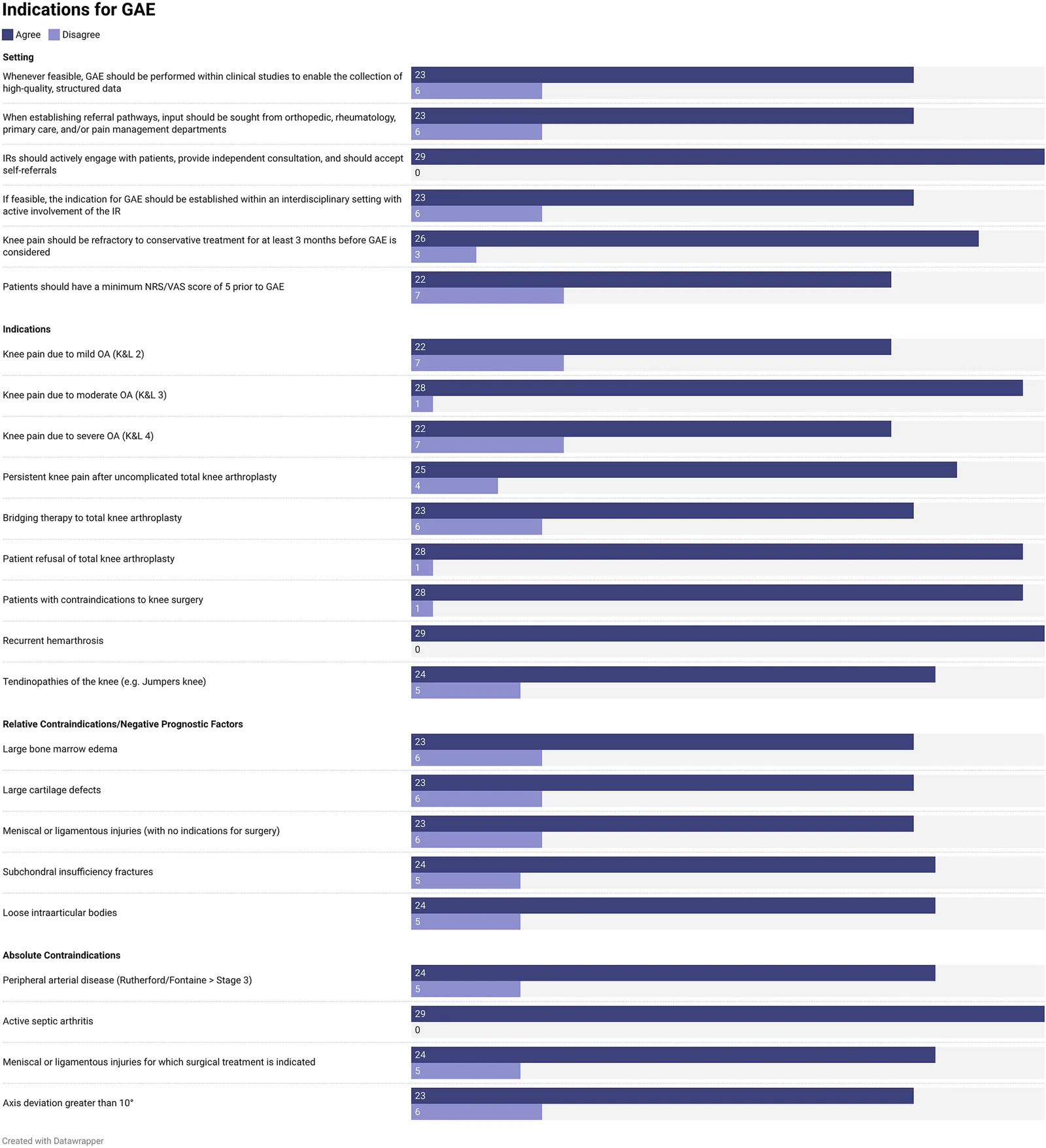
Consensus results for patient selection in GAE. Bar chart displaying the number of panelists agreeing and disagreeing with each statement across four domains: setting, indications, relative contraindications/negative prognostic factors, and absolute contraindications. GAE: Genicular artery embolization; IR: Interventional radiologist; K&L: Kellgren–Lawrence grade: NRS: Numeric rating scale; VAS: Visual analog scale

**Fig. 2:**
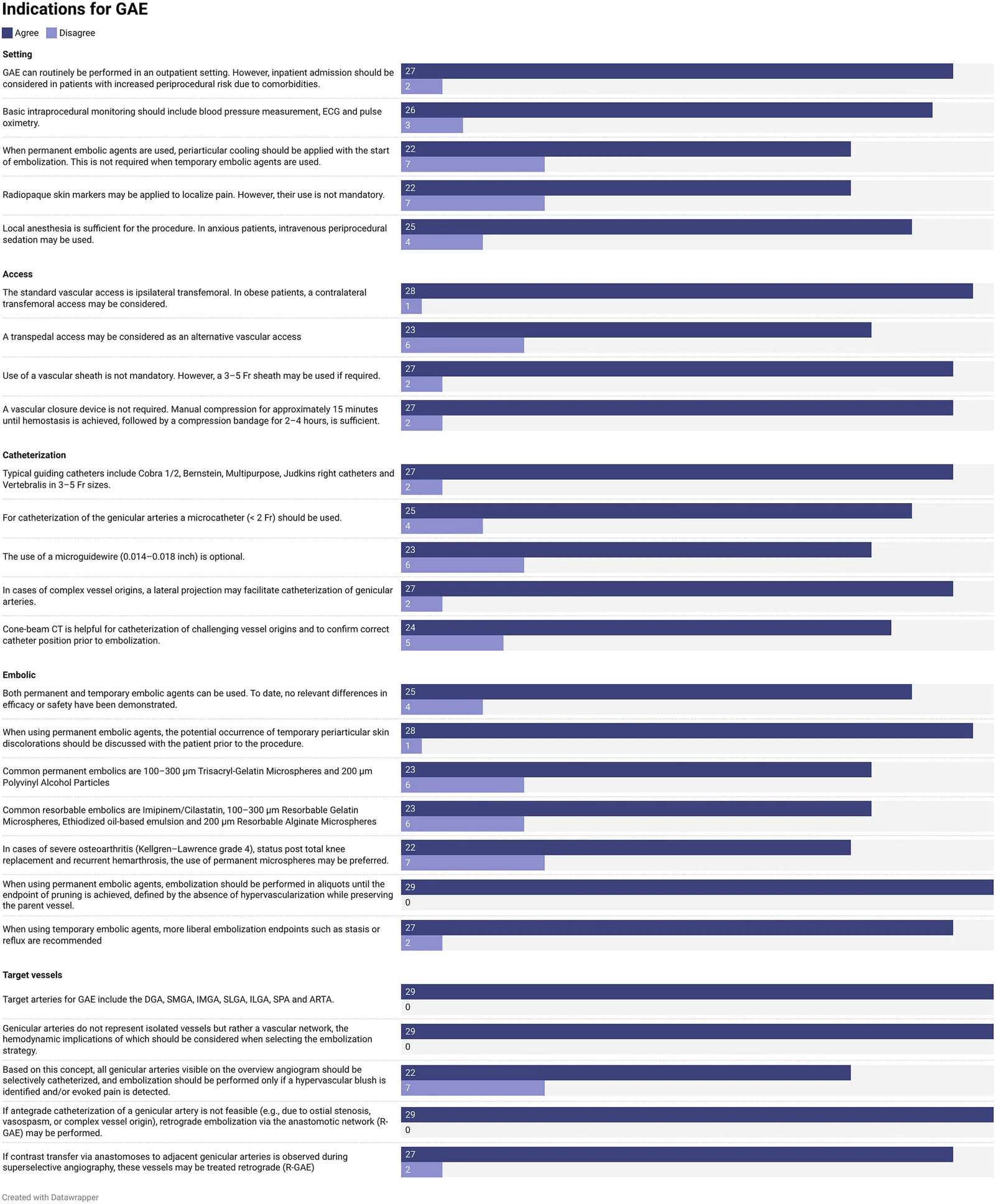
Consensus results for procedural technique in GAE. Bar chart displaying the number of panelists agreeing and disagreeing with each statement across four domains: setting, access, catheterization, embolic agent selection and target vessels.: GAE: Genicular Artery Embolization; BP: Blood Pressure; ECG: Electrocardiogram; Fr: French; CB-CT: Cone-Beam CT; DGA: Descending Genicular Artery; SMGA: Superior Medial Genicular Artery; IMGA: Inferior Medial Genicular Artery; SLGA: Superior Lateral Genicular Artery; ILGA: Inferior Lateral Genicular Artery; SPA: Superior Patellar Artery; ARTA: Anterior Recurrent Tibial Artery; R-GAE: Retrograde Genicular Artery Embolization; OA: Osteoarthritis; TKA: Total Knee Arthroplasty

**Fig. 3:**
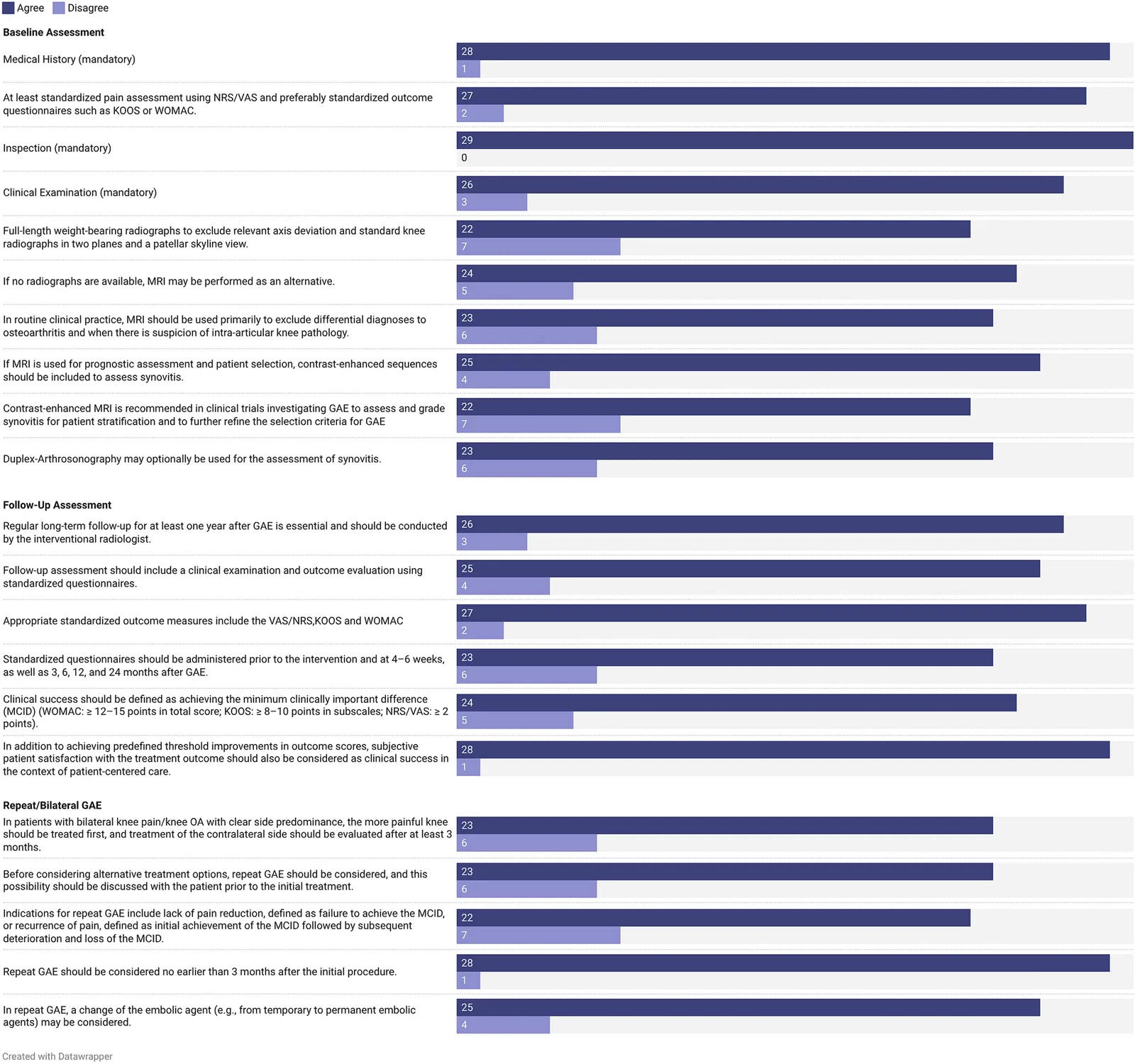
Consensus results for clinical care in GAE. Bar chart displaying the number of panelists agreeing and disagreeing with each statement across three domains: baseline assessment, follow-up assessment and repeat/bilateral GAE. GAE: Genicular artery embolization; K&L: Kellgren–Lawrence Grade; KOOS: Knee injury and osteoarthritis outcome score; MCID: Minimal clinically important difference; NRS: Numeric rating scale; OA: Osteoarthritis; VAS: Visual analog scale; WOMAC: Western Ontario and McMaster universities osteoarthritis index

All 29 panelists completed all three rounds (Table 1). Consensus was reached on 71 statements, while 4 did not meet consensus. Kendall's W increased between the second and third round (W = 0.267 vs. 0.325; *p* ≤ *0.0001*). Percentage agreement for each statement is presented in Figs. 1, 2 and 3.

**Table 1:** Characteristics of panelists

*Country of practice, n (%)*	8 (28%)
USA	5 (17%)
Germany	3 (10%)
Brazil	2 (7%)
UK	1 (3%)
France	1 (3%)
Greece	1 (3%)
Italy	1 (3%)
Spain	1 (3%)
Portugal	1 (3%)
Switzerland	1 (3%)
Turkey	1 (3%)
Egypt	1 (3%)
India	1 (3%)
Japan	1 (3%)
Australia	1 (3%)
*Fields of Expertise, n (%)*	
Embolization	29 (100%)
Arterial Intervention	22 (76%)
Interventional Oncology (IO)	20 (69%)
Non-Vascular Interventions	17 (59%)
Venous Interventions	17 (59%)
Aortic Intervention	9 (31%)
Pediatric Interventions	7 (24%)
Neurointervention	3 (10%)
Experience in IR, years median (range)	14 (4–36)
Experience in GAE, years median (range)	4 (1–13)
Number of GAEs performed, median (range)	160 (50–1500)
H-Index, median (range)	16 (3–45)

### Indications for GAE

Of 27 statements addressing indications, 22 reached consensus in round two (median 9[IQR 7–10]; 83% ≥ 7) and two additional statements in round three (8[8–10]; 81% ≥ 7; *p* ≤ *0.05*); three statements failed to reach consensus (2[1–6.5]; 24% ≥ 7; *p* > *0.05*) (Supplementary Table 2; Fig. 1). Consensus was reached that GAE should be performed within clinical studies whenever feasible to enable structured collection of high-quality data and that departments of orthopedics, rheumatology, primary care and pain management should be approached when establishing referral pathways. Full agreement was reached that IRs should play an active role in interdisciplinary indication setting and independent patient consultation.

Chronic knee pain refractory to conservative treatment for at least three months with a minimum numeric rating scale (NRS) score of 5, reached consensus as the minimum threshold for GAE candidacy. Indication without a minimum threshold did not reach consensus (2[1–5]; 21% ≥ 7).

Consensus indications include mild to severe radiographic osteoarthritis (K&L 2–4), tendinopathies of the knee (e.g., patellar tendinopathy), recurrent hemarthrosis, bridging therapy to total knee arthroplasty (TKA), patient refusal of or contraindication to TKA and persistent pain after uncomplicated TKA. Inflammatory arthritis (e.g., rheumatoid arthritis, psoriatic arthritis) did not reach consensus as an indication (4[2–7]; 24% ≥ 7).

Consensus relative contraindications associated with reduced outcomes for which GAE should be considered only after thorough patient counseling included, large bone marrow lesion, significant cartilage defects, meniscal or ligamentous injuries without surgical indication, subchondral insufficiency fractures and loose intra-articular bodies. In addition to standard contraindications to angiography, consensus absolute contraindications for GAE included peripheral arterial disease (Fontaine > 3/Rutherford > 4), active septic arthritis, meniscal or ligamentous injuries with surgical indication and axial deviation greater than 10°. Severe osteoarthritis was not considered a contraindication (5[1–8]; 48% ≥ 7).

### Technique

Procedural technique was addressed in 26 statements. Consensus was reached on all in round two (9[8–10]; 91% ≥ 7) (Supplementary Table 2; Fig. 2).

Consensus was reached that GAE can be performed in an inpatient or outpatient setting under local anesthesia, with intravenous sedation reserved for anxious patients. Periprocedural monitoring deemed necessary, includes blood pressure, ECG and pulse oximetry. An ipsilateral antegrade transfemoral approach reached consensus as the standard vascular access, with contralateral transfemoral and transpedal access as alternatives. A vascular sheath was not considered mandatory, and manual compression followed by compression bandage for 2–4h was regarded as sufficient for hemostasis.

For catheterization, consensus was reached on the use of 3–5 Fr guiding catheters (Cobra 1/2, Bernstein, Multipurpose, Judkins right), with a microcatheter smaller than 2 Fr for superselective catheterization. Use of a lateral projection and/or Cone-beam CT with vessel mapping were considered helpful for challenging vessel origins and confirmation of catheter position prior to embolization. Consensus target arteries include the descending genicular artery (GA), superomedial GA, inferomedial GA, superolateral GA, inferolateral GA, superior patellar artery and the anterior recurrent tibial artery. Consensus was reached that GAs should not be viewed as isolated vessels but rather as a vascular network and that all visible GAs should be selectively catheterized, with embolization performed upon detection of a hypervascular blush and/or evoked pain. Retrograde embolization via anastomoses (R-GAE) reached consensus as an approach when antegrade catheterization is not feasible or when contrast transfer to adjacent GAs via anastomoses is observed.

The panel reached consensus that permanent and temporary embolic agents are equally acceptable. Common permanent embolics identified by consensus were 100–300 µm trisacryl-gelatin microspheres (TAGM) and 200 µm polyvinyl alcohol particles (PVAP) while common resorbable agents were Imipenem/Cilastatin, 100–300 µm resorbable gelatin microspheres (RGM), ethiodized oil-based emulsion (EOE) and 200 µm resorbable alginate microspheres (RAM).

Consensus was reached that permanent microspheres may be preferred in radiographically severe osteoarthritis, status post-TKA or recurrent hemarthrosis. The endpoint of pruning, defined as absence of hypervascularization with preservation of the parent vessel, reached consensus as the appropriate embolization endpoint when using permanent agents. More liberal endpoints such as stasis or reflux were recommended when using temporary agents. The panel agreed that the potential for temporary periarticular skin discoloration with permanent embolics should be discussed with the patient prior to the procedure.

### Clinical Care

Clinical care was addressed in 22 statements. 21 reached consensus in round two (9[8–10]; 86% ≥ 7), while one statement did not reach consensus (8[5–9]; 66% ≥ 7; *p* > *0.05*), (Supplementary Table 2; Fig. 3).

Consensus baseline assessment included medical history, clinical examination, standardized pain assessment using NRS/VAS and preferably validated outcome questionnaires such as knee injury osteoarthritis outcome score (KOOS) and/or Western Ontario and McMaster Universities Osteoarthritis Index (WOMAC). Full-length weight-bearing radiographs, knee radiographs in two planes and a patellar skyline view reached consensus as useful. The panel agreed that in routine practice, MRI is primarily indicated to exclude differential diagnoses and that contrast-enhanced (CE-MRI) sequences should be included when MRI is used for prognostic assessment and patient selection, particularly in clinical trials. Duplex arthro-sonography reached consensus as an alternative tool for synovitis assessment.

The panel reached agreement that IRs should play an active role in longitudinal clinical care following GAE. Structured follow-up for at least one year was considered essential, comprising clinical examination and standardized outcome assessment at scheduled intervals. Clinical success reached consensus as achievement of the minimal clinically important difference (MCID; WOMAC: ≥ 12–15 points; KOOS-Pain: ≥ 8–10 points; NRS/VAS: ≥ 2 points) or individual patient satisfaction, defined as patient global assessment (PGA) score of ≥ 7 on a 10-point scale.

In patients with bilateral knee pain with clear side predominance, treating the more symptomatic knee first and evaluating the contralateral side after at least three months reached consensus. Treatment of both knees in a single session did not reach consensus. Discussing repeat GAE with the patient prior to the initial procedure, before considering alternative treatments, reached consensus. Consensus indications for repeat GAE included failure to achieve the MCID and pain recurrence following initial clinical success. Repeat GAE was considered to be appropriate no earlier than three months after the initial procedure, with consideration of a change of embolic agent (e.g., from temporary to permanent microspheres).

## Discussion

**Fig. 4:**
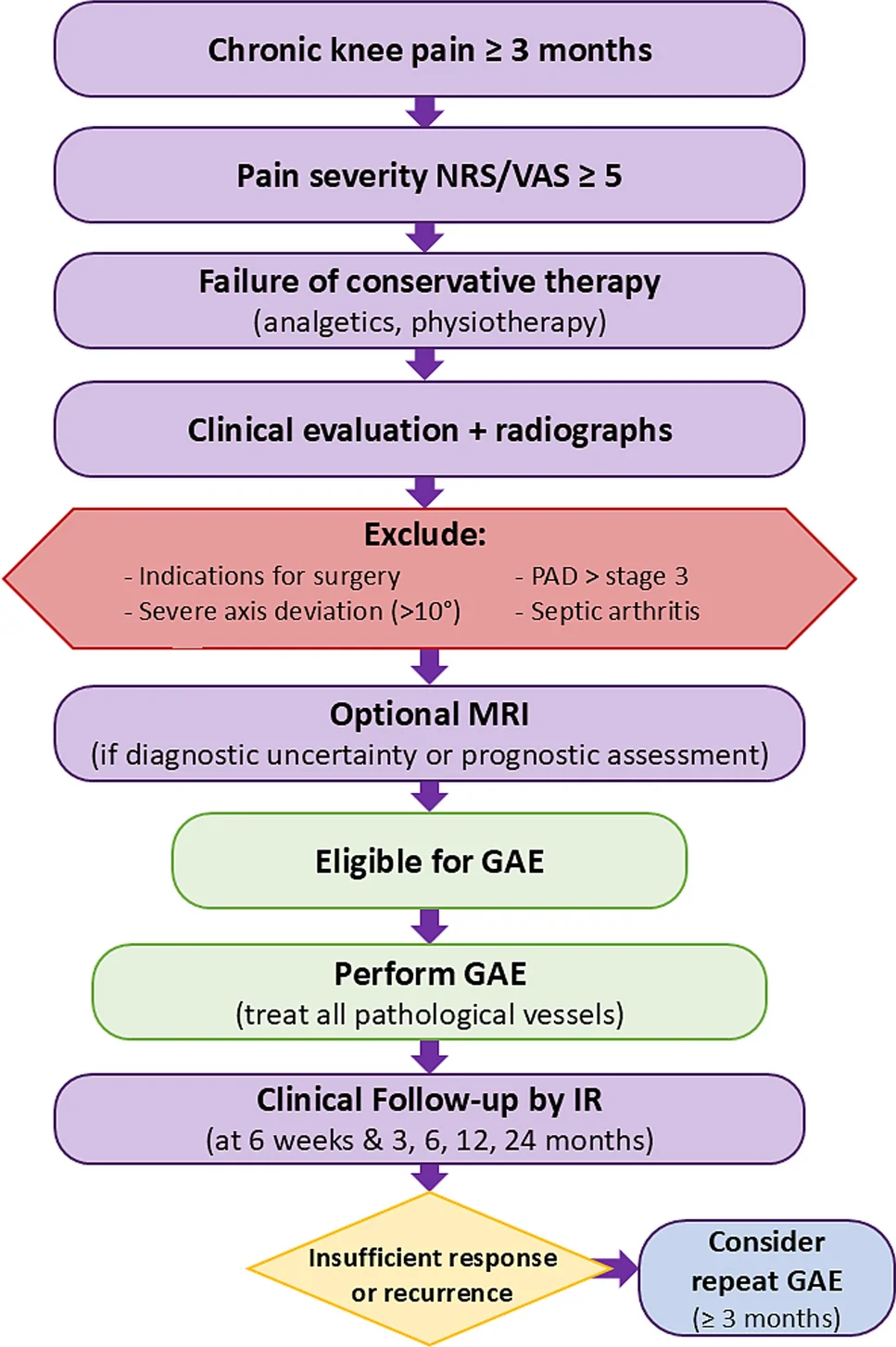
Treatment algorithm for genicular artery embolization

This study provides consensus-based expert recommendations on GAE with focus on patient selection, patient-tailored technique and longitudinal clinical care (Fig. 4; Tables 2, 3 and 4).

**Table 2:** Consensus recommendations for patient selection in GAE

Indications	Refractory knee pain for at least 3 months
	Minimum NRS/VAS score of 5
	Knee pain due to radiographically mild to severe OA (K&L 2–4)
	Persistent knee pain after uncomplicated total knee arthroplasty
	Bridging therapy to total knee arthroplasty
	Patients with contraindications to knee surgery
	Recurrent hemarthrosis
	Tendinopathies of the knee (e.g., Jumper's knee)
Relative contraindications/Negative prognostic factors (GAE should be considered only as a last resort and after appropriate patient counseling)	Large bone marrow edema
	Large cartilage defects
	Meniscal/Ligamentous injuries with no indication for surgery
	Subchondral insufficiency fractures
	Loose intraarticular bodies
Absolute contraindications (Besides standard contraindications to angiography)	Peripheral arterial disease (Fontaine > 3/Rutherford > 4)
	Active septic arthritis
	Meniscal/Ligamentous injuries with indication for surgery
	Axis deviation greater than 10°

All listed items achieved consensus (≥ 75% of ratings ≥ 7)

GAE: Genicular artery embolization; K&L: Kellgren–Lawrence grade; NRS: Numeric rating scale; OA: Osteoarthritis; VAS: Visual analog scale

**Table 3:** Consensus recommendations on technique in GAE

Setting	In an inpatient or outpatient setting
	Monitoring should include BP, ECG and pulse oximetry
	Periarticular cooling only necessary for permanent embolic
	Radiopaque skin markers for pain localization are not mandatory
	Local anesthesia is sufficient
Vascular Access	Standard: Ipsilateral transfemoral
	Alternatives: Contralateral transfemoral /Ipsilateral transpedal e.g., in obesepatients
	Vascular sheath (3–5 Fr) is not mandatory
	Vascular closure device is not required
Catheterization	Guiding catheters (3–5 Fr): Cobra 1/2, Bernstein, Multipurpose, Judkins right catheters and Vertebralis
	Microcatheter (< 2 Fr) should be used
	Microguidewire (0.014–0.018 in) is optional
	Lateral Projection / CB-CT for complex vessel origins
Target Vessels	Target vessels: DGA, SMGA, IMGA, SLGA, ILGA, SPA and ARTA
	All genicular arteries should be selectively catheterized and embolized upon detection of a hypervascular blush and/or evoked pain
	If antegrade GAE is not feasible or contrast transfer via anastomoses to adjacent genicular arteries is observed, consider retrograde GAE via the anastomotic network (R-GAE)
Choice of Embolic •	No evidence of superiority of either temporary or permanent embolic agents in terms of safety or efficacy has been demonstrated
	Common permanent embolics: 100–300 µm Trisacryl-Gelatin Microspheres and 200 µm Polyvinyl Alcohol Particles
	Common temporary embolics: Imipenem/Cilastatin, 100 300 µm Resorbable Gelatin Microspheres, Ethiodized Oil based. Emulsion, 200 µm Resorbable Algin ate Microspheres
	Embolization endpoint: pruning for permanent; stasis/ reflux for temporary
	Permanent agents may be preferred in severe OA, post-TKA pain, and recurrent hemarthrosis
	Temporary periarticular skin discoloration in permanent agents should be discussed with the patient prior to the procedure

All listed items achieved consensus (≥ 75% of ratings ≥ 7)

BP: Blood pressure; ECG: Electrocardiogram; Fr: French; CB-CT: Cone-beam computed tomography; DGA: Descending genicular artery; SMGA: Superomedial genicular artery; IMGA: Inferomedial genicular artery; SLGA: Superolateral genicular artery; ILGA: Inferolateral genicular artery; SPA: Superior patellar artery; ARTA: Anterior recurrent tibial artery; R-GAE: Retrograde genicular artery embolization; OA: Osteoarthritis

**Table 4:** Consensus recommendations on Clinical Care in GAE

Baseline Assessment	Mandatory: Medical History; Standardized pain assessment; Clinical Examination; Radiographs (Weight bearing & two planes)
	Optional: CE-MRI (to exclude differential diagnoses, prognostic assessment & patient selection especially in clinical trials), Duplex-Arthrosonography
Follow-Up	Clinical visits at 6 weeks, 3, 6, 12 and 24 months after GAE, performed by the Interventional Radiologist
	Appropriate outcome measures: VAS, NRS, KOOS, WOMAC
	Clinical success: Achievement of MCID and/or subjective patient satisfaction defined as Patient Global Assessment score of ≥ 7 on a 10-point scale
Bilateral /Repeat-GAE:	The more painful knee should be treated first and treatment of the contralateral side should be evaluated after at least 3 months
	Repeat GAE should be considered in patients with lack of pain reduction or recurrence of pain at least 3 months after the initial procedure
	In repeat GAE, consider change in the embolic agent (e.g., from temporary to permanent embolic agents)

All listed items achieved consensus (≥ 75% of ratings ≥ 7)

CE-MRI: Contrast-enhanced MRI; GAE: Genicular artery embolization; KOOS: Knee injury and osteoarthritis outcome score; MCID: Minimal clinically important difference; NRS: Numeric rating scale; OA: Osteoarthritis; VAS: Visual analog scale; WOMAC: Western Ontario and McMaster universities osteoarthritis index

When indicating GAE, pain history is essential. The threshold of an NRS ≥ 5 for at least three months has been adopted across prospective studies and is intended to ensure clinically meaningful benefit [3, 17, 18]. Below this threshold, pain is considered mild and achievement of MCID is unlikely [19, 20]. Beyond osteoarthritis and recurrent hemarthrosis, knee pain due to tendinopathies and after uncomplicated TKA may be effectively treated by GAE [1, 21, 22]. Advanced structural damage on MRI has been associated with reduced outcome, hence their classification as relative contraindications [23–25]. However, severe osteoarthritis is an accepted indication and clinical benefit has been demonstrated in this population [1, 9]. Absolute contraindications reflect secondary conditions requiring further treatment. To provide independent patient counseling, clinical evaluation is therefore essential and considered a core competency of IR.

To optimize procedural tolerability, non-ionic iso-osmolar contrast agents may be preferred over low-/high-osmolar formulations, as they are associated with less injection-related discomfort [26].

Technique significantly influences clinical outcome. The most outcome-relevant aspects are embolic selection and number of treated vessels [12]. Regarding the latter, GAs are increasingly viewed as a vascular network whose entire afferent supply must be addressed to achieve reduction of pathological hyperperfusion [11]. Accordingly, only catheterization and if necessary, embolization of *all* visible GA leads to a relevant clinical improvement [12]. A final angiography can help to identify accessory genicular arteries that recruit following embolization [1, 12, 27]. The anastomotic network is useful in GAE, as it allows treatment of multiple territories via a single GA [11]. Cone-beam CT may be considered to facilitate navigation [28]. Regarding embolic agent selection, a recently publish prospective and several retrospective studies have demonstrated comparable efficacy and safety between permanent and temporary agents [29–31]. While the largest evidence exists for Imipenem/Cilastatin, its use in GAE is off-label, as it is an antibiotic compound. In Europe, TAGM and PVA as permanent and RGM as temporary embolics, hold CE certification for GAE. In the USA, these and other embolics, including RAM and EOE have received either Investigational Device Exemption or Breakthrough Device designation and remain off-label pending regulatory approval. Embolization endpoints differ between permanent and temporary embolics, with pruning recommended for permanent and stasis/reflux for temporary agents [6, 17, 32]. No prospective comparative data are currently available to support recommendation of a specific embolic. Therefore, the proposed preference of permanent embolics in severe osteoarthritis, post-TKA pain and recurrent hemarthrosis currently reflects expert opinion rather than established evidence. Patients should be informed about the composition of embolics and potential risks prior to the procedure.

Adequate clinical care is another determinant of clinical success. IRs must be present as equal partners in interdisciplinary meetings, be available for independent patient consultation and play an active part in longitudinal clinical follow-up. Validated outcome questionnaires are essential to objectively quantify treatment response using established thresholds such as the MCID and PGA [14].

Pre-procedural imaging requirements differ between routine clinical practice and clinical trials. In clinical practice, radiographs are standard, while MRI is reserved for clinically ambiguous cases to exclude differential diagnoses and intra-articular pathology. Therefore MRI is not recommended as routine workup by current guidelines and is not obligatory prior to GAE [33, 34]. In clinical trials, CE-MRI is recommended to assess and grade synovitis, as it provides valuable prognostic information that can refine selection criteria for GAE [23, 24].

Longitudinal patient care after GAE encompasses the development of individualized long-term treatment strategies and if necessary, repeat interventions. In the open-ended first round most panelists (86%) preferred sequential treatment, allowing clinical response to the first procedure to guide the need for contralateral GAE, while a minority (14%) routinely performed single-session bilateral GAE for overload avoidance and reduced procedural burden. The efficacy of repeat and bilateral GAE has been demonstrated in initial studies, both performed no earlier than three months after the initial procedure, consistent with the recommended response-guided timing [35, 36].

This study has several limitations. Geographic representation was predominantly North American and European. However, the panel included key opinion leaders from all six continents, ensuring broad expertise. Inclusion criteria may have introduced selection bias and potential intellectual conflict of interest toward investigators already established in GAE. However, these criteria ensured that participating IRs had completed the learning curve and were up to date with the current research landscape. The risk of conflict of interest was further mitigated by the anonymized rating process. The recommendations represent expert consensus derived from an IR-only panel and multidisciplinary perspectives from other specialties involved in pain management remain needed. Areas without consensus reflect genuine clinical uncertainty and identify priorities for future investigation.

This study provides expert-derived consensus recommendations of an IR panel for patient selection, technique and clinical care in GAE. While many recommendations are supported by existing evidence, others are not yet supported by robust data. These recommendations require validation through prospective studies, with comparison of embolic agents, embolization endpoints, repeat GAE and optimal patient selection representing the most pressing research priorities.

## Supplementary Information

Below is the link to the electronic supplementary material.

Supplementary file1 (DOCX 52 kb)
