# Correction: Genicular Artery Embolization for Chronic Knee Pain: Expert Consensus Recommendations on Indications, Technique and Clinical Care Using a Delphi Process

**DOI:** 10.1007/s00270-026-04646-6

**Published:** 2026-10-01

**Authors:** A. Taheri Amin, J. Golzarian, K. Abd El Tawab, L. Abu-Gharbieh, O. Ahmed, A. Assis, S. A. Astani, CA. Binkert, Francisco Cesar Carnevale, F. Cavalheiro, F. Collettini, M. P. Correa, A. Dablan, K. Damodharan, Y. Epelboym, A. M. Fernández, D. Filippiadis, G. S. Goh, A. Guermazi, Z. Haskal, A. M. Ierardi, M. Katoh, M. Little, Y. Okuno, S. Padia, N. Rostambeigi, M. Sapoval, B. Taslakian, R. Uberoi, H. Vieweg, F. Ziayee, P. Minko

**Affiliations:** 1 Department of Diagnostic and Interventional Radiology, Charité Universitätsmedizin Berlin, Corporate Member of Freie Universität Berlin and Humboldt-Universität Zu Berlin, Berlin, Germany; 2 Department of Diagnostic and Interventional Radiology, Medical Faculty, University Hospital Duesseldorf, 40225 Duesseldorf, Germany; 3 Department of Radiology, University of Minnesota, Minneapolis, MN USA; 4 North Star Vascular Interventional Institute, Minneapolis, MN USA; 5 Department of Radiology, Interventional Radiology Unit, Ain Shams University Hospitals, Abbasia, Cairo, Egypt; 6 Joint and Vascular Institute, Libertyville, IL USA; 7 Department of Radiology, University of Utah, Salt Lake City, IL USA; 8 Interventional Radiology and Endovascular Surgery Department, University of Sao Paulo Medical School, Dr. Enéas de Carvalho Aguiar Avenue, São Paulo, Cerqueira César 25505403000 Brazil; 9 University of Minnesota, 420 Delaware St.SE, Mayo B228, RadiologyMinneapolis, MN 55455 USA; 10 Imagiology Department, Gaia Espinho Local Health Unit, V. N. de Gaia, Portugal; 11 Imagiology Department, CUF Porto Hospital, Porto, Portugal; 12 BIH Charité Clinician Scientist Program, BIH Biomedical Innovation Academy, Berlin Institute of Health, Charité-Universitätsmedizin Berlin, 10117 Berlin, Germany; 13 Instituto Vascular de Passo Fundo (Invasc), Passo Fundo, RS Brazil; 14 School of Medicine, Universidade de Passo Fundo, Passo Fundo, RS Brazil; 15 School of Medicine, Atitus Educação, Passo Fundo, RS Brazil; 16 Department of Radiology, Basaksehir Cam and Sakura City Hospital, Istanbul, Turkey; 17 Department of Vascular and Interventional Radiology, Madras Institute of Orthopedics and Traumatology (MIOT) Hospitals, Chennai, India; 18 Department of Radiology, Brigham and Women’s Hospital, Boston, MA USA; 19 Vascular and Interventional Radiology Department, University Hospital of León, Calle Altos de Nava, 24080 León, SN Spain; 20 Associate Professor of Diagnostic and Interventional Radiology, 2nd Department of Radiology, Medical School, University General Hospital “ATTIKON”, National and Kapodistrian University of Athens, Athens, Greece; 21 Department of Radiology, Alfred Health, Melbourne, VIC Australia; 22 Department of Surgery, School of Translational Medicine, Monash University, Victoria, Australia; 23 Quantitative Imaging Center, Department of Radiology, Boston University School of Medicine, 820 Harrison Avenue, FGH Building, 4Th Floor, Boston, MA 02118 USA; 24 Department of Radiology, VA Boston Healthcare System, 1400 VFW Parkway, , West Roxbury, MA 02132 USA; 25 Department of Radiology and Medical Imaging/Interventional Radiology. Professor of Radiology, University of Virginia School of Medicine, Charlottesville, USA; 26 Department of Diagnostic and Interventional Radiology, Foundation IRCCS Ca’ Granda Ospedale Maggiore Policlinico, 20122 Milan, Italy; 27 Department of Oncology and Hemato-Oncology, University of Milan, 20122 Milan, Italy; 28 Department of Diagnostic and Interventional Radiology, Helios Klinikum Krefeld, Lutherplatz, Krefeld, Germany; 29 University Department of Radiology, Royal Berkshire NHS Foundation Trust, Reading, UK; 30 University of Reading, Reading, UK; 31 Department of Interventional Radiology, Okuno Clinic Tokyo, Tokyo, Japan; 32 Department of Radiology, David Geffen School of Medicine, at the University of California Los Angels, Los Angeles, CA USA; 33 Vascular Interventional Radiology, Mallinckrodt Institute of Radiology, Washington University School of Medicine, St. Louis, MO 63110 USA; 34 Department of Vascular and Oncological Interventional Radiology, Hopital Europe´en Georges Pompidou, APHP, Paris, France; 35 Department of Radiology, NYU Langone Health, New York, USA; 36 Department of Radiology, John Radcliffe Hospital, Oxford University Hospitals NHS Trust, Headington, Oxford, OX3 9DU UK; 37 Faculty, Department of Radiology and Neuroradiology, Asklepios Klinik Nord, Academic Teaching Hospital of the University of Hamburg, Hamburg, Germany

**Correction to: Cardiovasc Intervent Radiol (2026) 49:1702–1714** **https://doi.org/10.1007/s00270-026-04547-8**

This article was updated to correct F. Carnevale to Francisco Cesar Carnevale in the author list.

