# Supplementary material for: Genicular Artery Embolization for Chronic Knee Pain: Expert Consensus Recommendations on Indications, Technique and Clinical Care Using a Delphi Process
Source: Cardiovasc Intervent Radiol. 2026 Jul 22;49(9):1702–14. doi: 10.1007/s00270-026-04547-8 (PMC13597583; doi:10.1007/s00270-026-04547-8)
Supplement: Supplementary file 1 [file 270_2026_4547_MOESM1_ESM.docx]

**Supplements**

**Supplementary Table 1:** **Complete results of the first open-ended round.**

Results of the first Delphi round, presented per question with response options, number of respondents selecting each option, and percentage relative to the full panel (n = 29). For questions allowing multiple selections, percentages may not sum to 100%. Of the 72 questions included in round one, 8 questions pertaining to panelist characterization (e.g., country of practice, field of expertise, experience in GAE) are not listed in this table. The corresponding results are presented in Table 1. Results of this exploratory round informed the development of the 75 statements rated in rounds 2 and 3.

*Abbreviations: GAE: Genicular Artery Embolization; IR: Interventional Radiologist; NRS: Numeric Rating Scale; VAS: Visual Analogue Scale; OA: Osteoarthritis; K&L: Kellgren–Lawrence Grade; TKA: Total Knee Arthroplasty; BMI: Body Mass Index; ECG: Electrocardiogram; Fr: French; DGA: Descending Genicular Artery; SMGA: Superomedial Genicular Artery; IMGA: Inferomedial Genicular Artery; SLGA: Superolateral Genicular Artery; ILGA: Inferolateral Genicular Artery; ARTA: Anterior Recurrent Tibial Artery; KOOS: Knee injury and Osteoarthritis Outcome Score; WOMAC: Western Ontario and McMaster Universities Osteoarthritis Index; MCID: Minimal Clinically Important Difference.*

| **No.** | **Question *(Answers)*** | **Responses** | **Percentage** |
| --- | --- | --- | --- |
| 1 | From which medical specialty are patients mainly referred to you? | | |
|  | *Radiology (radiology clinic)* | 9 | 31% |
|  | *Orthopedics* | 18 | 62% |
|  | *Rheumatology* | 5 | 17% |
|  | *Pain medicine* | 6 | 21% |
|  | *General practitioner* | 10 | 34% |
|  | *Self-referrals* | 21 | 72% |
| 2 | In what setting does the majority of your GAE procedures take place? | | |
|  | *Within a prospective study (including observational studies)* | *10* | *34%* |
|  | *Outside of a study* | *19* | *66%* |
| 3 | Who determines the indication for GAE? |  |  |
|  | *You as the interventional radiologist* | *20* | *69%* |
|  | *The referring physician* | *3* | *10%* |
|  | *Interdisciplinary conferences with IR and other specialties* | *6* | *21%* |
| 4 | What are indications for GAE? | | |
|  | *Knee pain due to osteoarthritis* | *29* | *100%* |
|  | *Knee pain due to tendinopathies* | *13* | *45%* |
|  | *Bridge to total knee arthroplasty* | *16* | *55%* |
|  | *Knee pain after total knee arthroplasty* | *22* | *76%* |
|  | *Knee pain related to inflammatory arthritis (e.g. rheumatoid arthritis, psoriatic arthritis)* | *7* | *24%* |
|  | *Recurrent hemarthrosis* | *21* | *72%* |
| 5 | For how long should a patient experience therapy-refractory pain to be considered a candidate for GAE? | | |
|  | *One month* | *1* | *3%* |
|  | *Three months* | *13* | *45%* |
|  | *Four months* | *1* | *3%* |
|  | *Six months* | *14* | *48%* |
| 6 | Is there a minimum pain severity (NRS/VAS) required for a patient to be considered for GAE?[ | | |
|  | *No minimum value; the indication for GAE is based on clinically relevant pain prompting the patient to seek medical treatment* | *14* | *48%* |
|  | *Yes, a minimum NRS/VAS score of is required...* | *15* | *52%* |
| 7 | Beyond the standard contraindications to angiography, what do you consider to be contraindications for GAE? | | |
|  | *Advanced knee OA (Kellgren & Lawrence Grade 4)* | *6* | *21%* |
|  | *Axis deviation greater than 10°* | *10* | *34%* |
|  | *Large Bone Marrow Lesions on MRI* | *5* | *17%* |
|  | *Meniscal tears* | *11* | *38%* |
|  | *Cruciate ligament ruptures* | *10* | *34%* |
|  | *Peripheral arterial disease* | *19* | *66%* |
| 8 | Which examinations do you personally perform when you see patients for the first time? | | |
|  | *Medical history* | *27* | *93%* |
|  | *Inspection* | *22* | *76%* |
|  | *Clinical examination* | *25* | *86%* |
|  | *Joint ultrasound* | *7* | *24%* |
| 9 | Which imaging modality is mandatory for you before GAE? | | |
|  | *X-ray* | *24* | *83%* |
|  | *CT* | *1* | *3%* |
|  | *MRI* | *17* | *59%* |
|  | *DVT* | *1* | *3%* |
|  | *Ultrasound* | *5* | *17%* |
| 10 | Setting of the intervention | | |
|  | *Outpatient (radiology day unit)* | *17* | *59%* |
|  | *Outpatient (external day unit)* | *6* | *21%* |
|  | *Inpatient (radiology ward)* | *4* | *14%* |
|  | *Inpatient (external ward)* | *2* | *7%* |
| 11 | Duration of the inpatient stay | | |
|  | *1 Night* | *6* | *21%* |
|  | *> 1 Night* | *0* | *0%* |
| 12 | Main reason for inpatient stay | | |
|  | *Billing reasons* | *6* | *21%* |
|  | *Comorbidities* | *1* | *3%* |
|  | *Peri-interventional complications* | *0* | *0%* |
| 13 | Anesthesia during the intervention | | |
|  | *Local anesthesia* | *26* | *90%* |
|  | *No i.v. sedation* | *4* | *14%* |
|  | *i.v. sedation* | *12* | *41%* |
|  | *No i.v. analgesia* | *3* | *10%* |
|  | *i.v. analgesia* | *5* | *17%* |
|  | *General anesthesia* | *1* | *3%* |
| 14 | Who administers the i.v. anesthesia during the intervention? | | |
|  | *Interventional radiologist* | *9* | *31%* |
|  | *Anesthesia team* | *2* | *7%* |
| 15 | What do you use for i.v. sedation / i.v. analgesia ? | | |
|  | *Midazolam* | *11* | *38%* |
|  | *Fentanyl* | *11* | *38%* |
|  | *Propofol* | *2* | *7%* |
| 16 | Periarticular cooling? | | |
|  | *Yes* | *12* | *41%* |
|  | *No* | *17* | *59%* |
| 17 | Commonly used access sites | | |
|  | *Ipsilateral transfemoral* | *28* | *97%* |
|  | *Contralateral transfemoral* | *9* | *31%* |
|  | *Transpedal* | *2* | *7%* |
| 18 | When do you use a contralateral transfemoral access site | | |
|  | *Obsese / High BMI* | *15* | *52%* |
|  | *Never* | *6* | *21%* |
|  | *Severe atherosclerotic disease* | *4* | *14%* |
| 19 | Introducer sheath | | |
|  | *Yes* | *22* | *76%* |
|  | *No* | *7* | *24%* |
| 20 | Introducer sheath size | | |
|  | *3 Fr* | *1* | *3%* |
|  | *4 Fr* | *14* | *48%* |
|  | *5 Fr* | *5* | *17%* |
|  | *6 Fr* | *1* | *3%* |
| 21 | Macrocatether | | |
|  | *Yes* | *28* | *97%* |
|  | *No* | *1* | *3%* |
| 22 | Configuration of Macrocatether | | |
|  | *Cobra 1 (C1)* | *3* | *10%* |
|  | *Cobra 2 (C2)* | *12* | *41%* |
|  | *Berenstein* | *11* | *38%* |
|  | *Multipurpose (MP-A1)* | *4* | *14%* |
|  | *Aachen Catheter* | *1* | *3%* |
|  | *Other (Vertebral; JR1-3; Internal mammary)* | *10* | *34%* |
| 23 | Do you use different macrocatheters for the catheterization of different genicular arteries? | | |
|  | *Yes* | *4* | *14%* |
|  | *No* | *24* | *83%* |
| 24 | Which catheter configuration do you typically use for the catheterization of each genicular artery (e.g. DGA, SMGA, IMGA, SLGA, ILGA, ARTA)? | | |
|  | *I will use a RIM catheter as a bailout when C2 fails* | *1* | *3%* |
| 25 | Catheter size | | |
|  | *3 Fr* | *2* | *7%* |
|  | *4 Fr* | *20* | *69%* |
|  | *5 Fr* | *4* | *14%* |
|  | *6 Fr* | *1* | *3%* |
| 26 | Microwire | | |
|  | *Yes* | *25* | *86%* |
|  | *No* | *4* | *14%* |
| 27 | Size of Microwire | | |
|  | *0.010″* | *0* | *0%* |
|  | *0.014″* | *16* | *55%* |
|  | *0.016″* | *7* | *24%* |
|  | *0.018″* | *1* | *3%* |
| 28 | Microcatheter | | |
|  | *Yes* | *28* | *97%* |
|  | *No* | *1* | *3%* |
| 29 | Size of Microcatheter | | |
|  | *1.7 Fr* | *15* | *52%* |
|  | *2.0 Fr* | *9* | *31%* |
| 30 | For the catheterization of which genicular arteries do you use a lateral projection? | | |
|  | *DGA* | *1* | *3%* |
|  | *SMGA* | *6* | *21%* |
|  | *IMGA* | *4* | *14%* |
|  | *SLGA* | *7* | *24%* |
|  | *ILGA* | *5* | *17%* |
|  | *I do not use a lateral projection* | *19* | *66%* |
| 31 | Closure of the access site | | |
|  | *Vascular closure device* | *5* | *17%* |
|  | *No vascular closure device* | *21* | *72%* |
|  | *Compression bandage* | *16* | *55%* |
| 32 | First choice of Embolic agent | | |
|  | *Embospheres* | *7* | *24%* |
|  | *Embozene* | *3* | *10%* |
|  | *Nexspheres* | *10* | *34%* |
|  | *Imipinem/Cilastatin* | *11* | *38%* |
|  | *Other (Lipiodol;SakuraBeads; Biopearls)* | *7* | *24%* |
| 33 | Do you use different embolic agents for different indications? | | |
|  | *Yes* | *10* | *34%* |
|  | *No* | *19* | *66%* |
| 34 | Which embolic agents do you use for which indications? | | |
|  | *Permanent in post-TKA pain* | *2* | *7%* |
|  | *Permanent in recurrent hemarthrosis* | *5* | *17%* |
| 35 | Preparation of the embolic material | | |
|  | *According to the manufacturer’s Instructions for Use (IFU)* | *19* | *66%* |
|  | *Deviation from the IFU (500mg IMP with 10 mL contrast; 3:1 Lipiodol:Optiray; Nexsphere: 3 NaCl : 7 CM; Diluted contrast and saline for a total volume of 20 mL)* | *8* | *28%* |
| 36 | Preferred embolic endpoint | | |
|  | *Pruning* | *19* | *66%* |
|  | *Stasis/Reflux* | *12* | *41%* |
| 37 | Do you use different embolic endpoints for different embolic agents? | | |
|  | *Yes* | *13* | *45%* |
|  | *No* | *16* | *55%* |
| 38 | Which embolic endpoints do you use for which embolic agents? | | |
|  | *Pruning for permanent, Stasis/Reflux for temporary* | *8* | *28%* |
|  | *Stasis/Reflux for Imipinem/Cilastatin* | *4* | *14%* |
| 39 | Do you place radiopaque markers at the patient’s point of maximal pain prior to the intervention? | | |
|  | *Yes* | *12* | *41%* |
|  | *No* | *17* | *59%* |
| 40 | Do you perform embolization only when a vascular blush is detected? | | |
|  | *Yes, I embolize only if a vascular blush is present* | *12* | *41%* |
|  | *No, I also embolize in the absence of a visible blush* | *8* | *28%* |
| 41 | In which situations do you perform embolization even in the absence of a visible vascular blush? | | |
|  | *I embolize all genicular arteries regardless of the presence of a blush* | *2* | *7%* |
|  | *If the genicular artery correlates with the patient’s pain localization, even without a visible blush* | *6* | *21%* |
|  | *If the genicular artery corresponds to the point of maximal synovitis or osteoarthritis on imaging, even without a visible blush* | *3* | *10%* |
| 42 | How do you select the arteries to be treated? | | |
|  | *Based on the patient’s pain localization* | *14* | *48%* |
|  | *Based on the point of maximal osteoarthritis or synovitis on radiography/MRI* | *7* | *24%* |
|  | *I treat all genicular arteries* | *18* | *62%* |
| 43 | What are the reasons for not embolizing all genicular arteries? | | |
|  | *Blush and/or pain/synovitis correlation as embolization criterion* | *5* | *17%* |
|  | *Anatomically based (collateralization makes individual treatment unnecessary)* | *1* | *3%* |
|  | *Technical minimum rule (at least one vessel per compartment)* | *3* | *10%* |
| 44 | Which genicular arteries do you treat in cases of medial knee pain? | | |
|  | *DGA* | *10* | *34%* |
|  | *SMGA* | *12* | *41%* |
|  | *IMGA* | *12* | *41%* |
|  | *SLGA* | *1* | *3%* |
|  | *ILGA* | *1* | *3%* |
| 45 | Which genicular arteries do you treat in cases of lateral knee pain? | | |
|  | *DGA* | *3* | *10%* |
|  | *SMGA* | *1* | *3%* |
|  | *IMGA* | *2* | *7%* |
|  | *SLGA* | *12* | *41%* |
|  | *ILGA* | *11* | *38%* |
|  | *ARTA* | *5* | *17%* |
| 46 | Which genicular arteries do you treat in cases of medial knee OA? | | |
|  | *DGA* | *6* | *21%* |
|  | *SMGA* | *6* | *21%* |
|  | *IMGA* | *6* | *21%* |
|  | *SLGA* | *1* | *3%* |
|  | *ILGA* | *1* | *3%* |
| 47 | Which genicular arteries do you treat in cases of lateral knee OA? | | |
|  | *DGA* | *3* | *10%* |
|  | *SMGA* | *1* | *3%* |
|  | *IMGA* | *1* | *3%* |
|  | *SLGA* | *7* | *24%* |
|  | *ILGA* | *7* | *24%* |
|  | *ARTA* | *4* | *14%* |
| 48 | If you state that you embolize all genicular arteries, please specify which genicular arteries are included. | | |
|  | *DGA* | *17* | *59%* |
|  | *SMGA* | *17* | *59%* |
|  | *IMGA* | *17* | *59%* |
|  | *SLGA* | *17* | *59%* |
|  | *ILGA* | *18* | *62%* |
|  | *ARTA* | *11* | *38%* |
| 49 | Please specify other indications and the corresponding genicular arteries you particulary embolize. | | |
|  | *No indication-specific vessel selection (treat all visible/blush-positive vessels regardless of indication)* | *9* | *31%* |
|  | *Patellofemoral pain / patellar tendinopathy → Superior Patellar Artery and/or ILGA* | *5* | *17%* |
| 50 | Is structured follow-up performed at regular intervals? | | |
|  | *Yes* | *27* | *93%* |
|  | *No* | *2* | *7%* |
| 51 | Who is responsible for the follow-up care of most patients? | | |
|  | *You as the interventional radiologist* | *26* | *90%* |
|  | *The referring physician* | *1* | *3%* |
| 52 | Up to what time period is follow-up care carried out? | | |
|  | *<1 Year* | *13* | *45%* |
|  | *> 1 Year* | *14* | *48%* |
| 53 | What is part of the structured follow-up? | | |
|  | *Standardized Questionnaires* | *24* | *83%* |
|  | *Pain history* | *25* | *86%* |
|  | *Clinical examination* | *19* | *66%* |
|  | *Imaging* | *7* | *24%* |
|  | Which questionnaires do you use for outcome assessment? | | |
|  | *NRS* | *4* | *14%* |
|  | *VAS* | *20* | *69%* |
|  | *KOOS* | *9* | *31%* |
|  | *WOMAC* | *18* | *62%* |
| 54 | At which time points do you collect outcome questionnaires? | | |
|  | *≤2 weeks (1 day / 1 week / 2 weeks)* | *5* | *17%* |
|  | *6 weeks* | *2* | *7%* |
|  | *1 month* | *8* | *28%* |
|  | *3 months* | *7* | *24%* |
| 55 | How do you define clinical success after GAE? | | |
|  | *Achievement of the Minimum Clinically Important Difference (WOMAC: ≥ 12–15 pt. in total score; KOOS: ≥ 8–10 pt. in subscales; NRS/VAS: ≥ 2 pt.)* | *12* | *41%* |
|  | *Reduction of pain by at least 50%* | *13* | *45%* |
|  | *Achievement of pain-free status* | *1* | *3%* |
| 56 | How do you manage patients with bilateral knee osteoarthritis? | | |
|  | *I treat both knees in a single session* | *4* | *14%* |
|  | *I treat one knee first and then re-evaluate the patient before treating the contralateral side* | *25* | *86%* |
| 57 | What is your rationale for treating both knees simultaneously? | | |
|  | *Biomechanical/compensatory overload rationale (untreated contralateral knee leads to compensatory overload of the treated knee)* | *1* | *3%* |
|  | *Reduced patient/procedural burden (treatment time, repeated hospital visits, procedure-related anxiety)* | *1* | *3%* |
|  | *Cost/reimbursement rationale* | *1* | *3%* |
| 58 | In patients with bilateral knee osteoarthritis, which side do you treat first? | | |
|  | *The side with more severe osteoarthritis/synovitis on imaging* | *4* | *14%* |
|  | *The side with more severe pain* | *21* | *72%* |
| 59 | At what earliest time point do you evaluate the contralateral knee for GAE? | | |
|  | *≤2 weeks* | *3* | *10%* |
|  | *4-6 weeks* | *3* | *10%* |
|  | *8 weeks* | *2* | *7%* |
|  | *1 month* | *7* | *24%* |
|  | *3 months* | *6* | *21%* |
|  | *6 months* | *1* | *3%* |
|  | *Immediate / at first presentation* | *1* | *3%* |
| 60 | Do you perform a repeat GAE? | | |
|  | *Yes* | *26* | *90%* |
|  | *No* | *3* | *10%* |
| 61 | At what earliest time point (in months) would you consider repeat GAE after the initial procedure? | | |
|  | *3 months* | *6* | *21%* |
|  | *2–3 months* | *1* | *3%* |
|  | *>3 months* | *1* | *3%* |
|  | *6 months* | *8* | *28%* |
|  | *12 months* | *7* | *24%* |
| 62 | What do you consider indications for repeat GAE? | | |
|  | *Lack of pain reduction after initial GAE* | *8* | *28%* |
|  | *Recurrence of pain after initial pain relief* | *25* | *86%* |
| 63 | What is the maximum number of GAE procedures you would perform on the same knee? | | |
|  | *Maximum of 2* | *12* | *41%* |
|  | *Maximum of 3* | *4* | *14%* |
|  | *No defined maximum* | *4* | *14%* |
| 64 | Do you use different embolic agents for repeat Genicular Artery Embolization (repeat GAE)? If yes, which combinations of embolic agents do you use, and what is the rationale for this approach? | | |
|  | *Yes – switches to permanent/more durable agent upon recurrence* | *16* | *55%* |
|  | *No – same agent used at repeat GAE* | *5* | *17%* |

**Supplementary Table 2: Complete Delphi results for Round two and three**

Results of the second and third Delphi rounds, presented per statement and grouped by domain and subdomain. For each statement, the median, minimum, maximum, interquartile range (IQR), and the number (%) of ratings ≥7 and ≤7 are reported (n = 29 panelists). Statements that reached consensus (≥75% of ratings ≥7) in round 2 are listed once. Statements that did not reach consensus in round two were resubmitted in round three, accompanied by the percentage agreement from round two. Results of both rounds are shown sequentially (1: round two; 2: round three), together with the p-value for the change in ratings between rounds (Wilcoxon signed-rank test for paired ordinal data).

*Abbreviations: GAE: Genicular Artery Embolization; IR: Interventional Radiologist; CI: Contraindication; OA: Osteoarthritis; K&L: Kellgren–Lawrence Grade; NRS: Numeric Rating Scale; VAS: Visual Analogue Scale; IQR: Interquartile Range; Fr: French; DGA: Descending Genicular Artery; SMGA: Superomedial Genicular Artery; IMGA: Inferomedial Genicular Artery; SLGA: Superolateral Genicular Artery; ILGA: Inferolateral Genicular Artery; SPA: Superior Patellar Artery; ARTA: Anterior Recurrent Tibial Artery; R-GAE: Retrograde Genicular Artery Embolization; KOOS: Knee injury and Osteoarthritis Outcome Score; WOMAC: Western Ontario and McMaster Universities Osteoarthritis Index; MCID: Minimal Clinically Important Difference.***Indications**

| **No.** | **Domain** | **Subdomain** | **Statement** | **Median** | **Minimum** | **Maximum** | **IQR** | **n ≥ 7**  (%) | **n ≤ 7**  (%) | |
| --- | --- | --- | --- | --- | --- | --- | --- | --- | --- | --- |
| 1 | Indications | Setting | Wherever feasible, GAE should be performed within clinical studies to enable the collection of high-quality, structured data. | 9 | 1 | 10 | 7-10 | 23  (79%) | 6  (21%) | |
| 2 | Indications | Setting | When establishing referral pathways, input should be sought from orthopedic, rheumatology, primary care, and/or pain management | 9 | 0 | 10 | 8-10 | 23  (79%) | 6  (21%) | |
| 3 | Indications | Setting | IRs, as clinically responsible physicians, should actively engage with patients, provide independent consultation, and should accept self-referrals | 10 | 7 | 10 | 10-10 | 29  (100%) | 0  (0%) | |
| 4 | Indications | Setting | If feasible, the indication for GAE should be established within an interdisciplinary setting with active involvement of the IR | 10 | 0 | 10 | 7-10 | 23  (79%) | 6  (21%) | |
| 5 | Indications | Setting | Knee pain should be refractory to conservative treatment for at least 3 months before GAE is considered. | 10 | 5 | 10 | 8-10 | 26  (90%) | 3  (10%) | |
| 6 (1) | Indications | Setting | There is no minimum NRS/VAS pain score that a patient must meet.  *(p=1)* | 2 | 0 | 10 | 1-6 | 7  (24%) | 21  (76%) | |
| 6 (2) |  |  |  | 2 | 0 | 10 | 1-6 | 7  (24%) | 21  (76%) | |
| 7 | Indications | Setting | Patients should have a minimum NRS/VAS score of 5 prior to GAE. | 8 | 0 | 10 | 7-10 | 22  (76%) | 7  (24%) | |
| 8 | Indications | Indications | Knee pain due to mild OA (K&L 2) | 9 | 1 | 10 | 7.5-10 | 22  (76%) | 7  (24%) | |
| 9 | Indications | Indications | Knee pain due to moderate OA (K&L 3) | 10 | 5 | 10 | 9.8-10 | 28  (97%) | 1  (3%) | |
| 10 | Indications | Indications | Knee pain due to severe OA (K&L 4) | 8 | 2 | 10 | 6.5-10 | 22  (76%) | 7  (24%) | |
| 11 | Indications | Indications | Persistent knee pain after total knee arthroplasty | 9 | 5 | 10 | 7-10 | 25  (86%) | 4  (14%) | |
| 12 | Indications | Indications | Bridging therapy to total knee arthroplasty | 8 | 3 | 10 | 7-10 | 23  (79%) | 6  (21%) | |
| 13 | Indications | Indications | Patient refusal of total knee arthroplasty | 10 | 5 | 10 | 8-10 | 28  (97%) | 1  (3%) | |
| 14 | Indications | Indications | Patients who are not surgical candidates due to significant comorbidities. | 10 | 5 | 10 | 8-10 | 28  (97%) | 1  (3%) | |
| 15 | Indications | Indications | Recurrent hemarthrosis | 10 | 8 | 10 | 10-10 | 29  (100%) | 0  (0%) | |
| 16 | Indications | Indications | Tendinopathies of the knee (e.g. Jumpers knee) | 9 | 1 | 10 | 7.8-10 | 24  (83%) | 5  (17%) | |
| 17  (1) | Indications | Indications | Inflammatory arthritis (e.g. Rheumatoid Arthritis, Psoriatic Arthritis)  *(p=1)* | 4 | 0 | 8 | 2-6.5 | 7  (24%) | 19  (76%) | |
| 17 (2) |  |  |  | 4 | 0 | 8 | 2-6.5 | 7  (24%) | 19  (76%) | |
| 18 | Indications | Relative CI | Large bone marrow edema | 8 | 3 | 10 | 7-10 | 23  (79%) | 6  (21%) | |
| 19 | Indications | Relative CI | Large cartilage defects | 8 | 3 | 10 | 7-10 | 23  (79%) | 6  (21%) | |
| 20  (1) | Indications | Relative CI | Meniscal or ligamentous injuries (with no indications for surgery)  *(p=0.04)* | 7 | 3 | 10 | 6-8 | 16  (55%) | 12  (45%) | |
| 20  (2) |  |  |  | 8 | 3 | 10 | 7-9 | 23  (79%) | 6  (21%) | |
| 21 | Indications | Relative CI | Subchondral insufficiency fractures | 9 | 1 | 10 | 7.8-10 | 24  (83%) | 5  (17%) | |
| 22  (1) | Indications | Relative CI | Loose intraarticular bodies  *(p=0.04)* | 7 | 3 | 10 | 6-8 | 16  (55%) | 12  (45%) | |
| 22  (2) |  |  |  | 9 | 1 | 10 | 8-10 | 24  (83%) | 5  (17%) | |
| 23 | Indications | Absolute CI | peripheral arterial disease (Fontaine>3/Rutherford>4) | 9 | 0 | 10 | 7-10 | 24  (83%) | 5  (17%) | |
| 24 | Indications | Absolute CI | Active septic arthritis | 10 | 7 | 10 | 10-10 | 29  (100%) | 0  (0%) | |
| 25 | Indications | Absolute CI | Meniscal or ligamentous injuries for which surgical treatment is indicated | 8 | 1 | 10 | 7-10 | 24  (83%) | 5  (17%) | |
| 26 | Indications | Absolute CI | Axis deviation greater than 10° | 8 | 1 | 10 | 7-8 | 24  (83%) | 5  (17%) | |
| 27  (1) | Indications | Absolute CI | Knee pain due to end-stage OA (K&L 4)  *(p=1)* | 5 | 0 | 10 | 1-8 | 14  (48%) | | 15  (52%) |
| 27  (2) |  |  |  | 5 | 0 | 10 | 1-8 | 14  (48%) | | 15  (52%) |

**Technique**

| **No.** | **Domain** | **Subdomain** | **Statement** | **Median** | **Minimum** | **Maximum** | **IQR** | **n ≥ 7**  (%) | **n ≤ 7**  (%) |
| --- | --- | --- | --- | --- | --- | --- | --- | --- | --- |
| 28 | Technique | Setting | GAE can routinely be performed in an outpatient setting; however, inpatient admission should be considered in patients with increased periprocedural risks due to comorbidities. | 10 | 0 | 10 | 8.8-10 | 27  (93%) | 2  (7%) |
| 29 | Technique | Setting | Basic intraprocedural monitoring includes blood pressure measurement, ECG and pulse oximetry. | 10 | 0 | 10 | 8-10 | 26  (90%) | 3  (10%) |
| 30 | Technique | Setting | When permanent embolic agents are used, periarticular cooling should be applied with the start of embolization; this is not required | 8 | 1 | 10 | 6.8-10 | 22  (76%) | 7  (24%) |
| 31 | Technique | Setting | Radiopaque skin markers may be applied to localize pain. However, their use is not mandatory. | 10 | 1 | 10 | 7.5-10 | 22  (76%) | 7  (24%) |
| 32 | Technique | Setting | Local anesthesia is sufficient for the procedure. In anxious patients, intravenous periprocedural sedation may be used. | 10 | 0 | 10 | 8-10 | 25  (86%) | 4  (14%) |
| 33 | Technique | Access | The standard vascular access is ipsilateral transfemoral; in obese patients, a contralateral transfemoral access may be considered. | 10 | 8 | 10 | 9-10 | 29  (100%) | 0  (0%) |
| 34 | Technique | Access | A transpedal access may be considered as an alternative vascular | 8 | 1 | 10 | 7-10 | 23  (79%) | 6  (21%) |
| 35 | Technique | Access | Use of a vascular sheath is not mandatory. However, a 3–5 Fr sheath may be used if required. | 10 | 6 | 10 | 8.8-10 | 27  (93%) | 2  (7%) |
| 36 | Technique | Access | A vascular closure device is not required. Manual compression for approximately 15 minutes until hemostasis is achieved, followed by a compression bandage for 2–4 hours, is sufficient. | 8 | 1 | 10 | 8.8-10 | 23  (79%) | 6  (21%) |
| 37 | Technique | Catheterization | Typical guiding catheters include Cobra 1/2, Bernstein, Multipurpose, Judkins right catheters and Vertebralis: typically in 3–5 Fr sizes. | 10 | 4 | 10 | 8-10 | 27  (93%) | 2  (7%) |
| 38 | Technique | Catheterization | For catheterization of the genicular arteries a microcatheter (< 2 Fr) should be used. | 10 | 1 | 10 | 8-10 | 25  (86%) | 4  (14%) |
| 39 | Technique | Catheterization | The use of a microguidewire (0.014–0.018 inch) is optional. | 8 | 1 | 10 | 7-10 | 23  (79%) | 6  (21%) |
| 40 | Technique | Catheterization | In cases of complex vessel origins, a lateral projection may facilitate catheterization of genicular arteries. | 10 | 6 | 10 | 8.8-10 | 27  (93%) | 2  (7%) |
| 41 | Technique | Catheterization | Cone-beam CT is helpful for catheterization of challenging vessel origins and to confirm correct catheter position prior to embolization. | 9 | 0 | 10 | 7.8-10 | 25  (86%) | 4  (14%) |
| 42 | Technique | Embolic | Both permanent and temporary embolic agents can be used. To date, no relevant differences in efficacy or safety have been demonstrated. | 10 | 0 | 10 | 8-10 | 25  (86%) | 4  (14%) |
| 43 | Technique | Embolic | When using permanent embolic agents, the potential occurrence of temporary periarticular skin discolorations should be discussed with | 10 | 6 | 10 | 9-10 | 28  (97%) | 1  (3%) |
| 44 | Technique | Embolic | Common permanent embolics are 100–300 µm Trisacryl-Gelatin Microspheres and 200 µm Polyvinyl Alcohol Particles | 10 | 0 | 10 | 7.3-10 | 23  (79%) | 6  (21%) |
| 45 | Technique | Embolic | Common resorbable embolics are Imipenem/Cilastatin, 100–300 µm Resorbable Gelatin Microspheres, Ethiodized oil-based emulsion and 200 µm Resorbable Alginate Microspheres | 9 | 0 | 10 | 7-10 | 23  (79%) | 6  (21%) |
| 46 | Technique | Embolic | In cases of severe osteoarthritis (Kellgren–Lawrence Grade 4), status post total knee replacement, and recurrent hemarthrosis, the use of permanent microspheres may be preferred. | 9 | 0 | 10 | 6.8-10 | 22  (76%) | 7  (24%) |
| 47 | Technique | Embolic | When using permanent embolic agents, embolization should be performed in aliquots until the endpoint of pruning is achieved, defined by the absence of hypervascularization while preserving the parent | 10 | 7 | 10 | 9-10 | 29  (100%) | 0  (0%) |
| 48 | Technique | Embolic | When using temporary embolic agents, more liberal embolization endpoints such as stasis or reflux are recommended. | 10 | 0 | 10 | 9-10 | 27  (93%) | 2  (7%) |
| 49 | Technique | Target Vessels | Target arteries for GAE include the descending genicular artery (DGA), superomedial genicular artery (SMGA), inferomedial genicular artery (IMGA), superolateral genicular artery (SLGA), inferolateral genicular artery (ILGA), Superior patellar artery (SPA) and the anterior recurrent tibial | 10 | 8 | 10 | 9-10 | 29  (100%) | 0  (0%) |
| 50 | Technique | Target Vessels | Genicular arteries do not represent isolated vessels but rather a vascular network; the hemodynamic implications of which should be considered when selecting the embolization strategy. | 10 | 8 | 10 | 9-10 | 29  (100%) | 0  (0%) |
| 51 | Technique | Target Vessels | Based on this concept, all genicular arteries visible on the overview angiogram should be selectively catheterized, and embolization should be performed only if a hypervascular blush is identified and/or evoked pain is detected. | 9 | 1 | 10 | 6.8-10 | 22  (76%) | 7  (24%) |
| 52 | Technique | Target Vessels | If antegrade catheterization of a genicular artery is not feasible (e.g., due to ostial stenosis, vasospasm, or complex vessel origin), retrograde embolization via the anastomotic network (R-GAE) may be performed. | 10 | 7 | 10 | 9-10 | 29  (100%) | 0  (0%) |
| 53 | Technique | Target Vessels | If contrast transfer via anastomoses to adjacent genicular arteries is observed during superselective angiography, these vessels may be treated retrograde (R-GAE) | 9 | 1 | 10 | 8-10 | 27  (93%) | 2  (7%) |

**Clinical Care**

| **No.** | **Domain** | **Subdomain** | **Statement** | **Median** | **Minimum** | **Maximum** | **IQR** | **n ≥ 7**  (%) | **n ≤ 7**  (%) |
| --- | --- | --- | --- | --- | --- | --- | --- | --- | --- |
| 54 | Clinical Care | Baseline Assessment | Medical History (mandatory) | 10 | 8 | 10 | 10-10 | 29  (100%) | 0  (0%) |
| 55 | Clinical Care | Baseline Assessment | At least standardized pain assessment using NRS/VAS and preferably standardized outcome questionnaires such as KOOS or WOMAC. | 10 | 5 | 10 | 8.8-10 | 27  (93%) | 2  (7%) |
| 56 | Clinical Care | Baseline Assessment | Inspection (mandatory) | 10 | 4 | 10 | 8-10 | 28  (97%) | 1  (3%) |
| 57 | Clinical Care | Baseline Assessment | Clinical Examination (mandatory) | 10 | 4 | 10 | 8-10 | 26  (90%) | 3  (10%) |
| 58 | Clinical Care | Baseline Assessment | Full-length weight-bearing radiographs to exclude relevant axis deviation, and standard knee radiographs in two planes and a patellar skyline view. | 8 | 2 | 10 | 7-10 | 22  (76%) | 7  (24%) |
| 59 | Clinical Care | Baseline Assessment | If no radiographs are available, MRI may be performed as an alternative. | 8 | 2 | 10 | 7-10 | 24  (83%) | 5  (17%) |
| 60 | Clinical Care | Baseline Assessment | In routine clinical practice, MRI should be used primarily to exclude differential diagnoses to osteoarthritis and when there is suspicion of intra-articular knee pathology. | 8 | 0 | 10 | 7-10 | 23  (79%) | 6  (21%) |
| 61 | Clinical Care | Baseline Assessment | If MRI is used for prognostic assessment and patient selection, contrast-enhanced sequences should be included to assess synovitis. | 9 | 3 | 10 | 8-10 | 25  (86%) | 4  (14%) |
| 62 | Clinical Care | Baseline Assessment | Contrast-enhanced MRI is recommended in clinical trials investigating GAE to assess and grade synovitis for patient stratification and to further refine the selection criteria for GAE | 8 | 3 | 10 | 7-9 | 22  (76%) | 7  (24%) |
| 63 | Clinical Care | Baseline Assessment | Duplex-Arthrosonography may optionally be used for the assessment of synovitis | 8 | 0 | 10 | 7-9.3 | 23  (79%) | 6  (21%) |
| 64 | Clinical Care | Follow-Up Assessment | Regular long-term follow-up for at least one year after GAE is essential and should be conducted by the interventional radiologist. | 10 | 3 | 10 | 8-10 | 23  (79%) | 6  (21%) |
| 65 | Clinical Care | Follow-Up Assessment | Follow-up assessment should include a clinical examination and outcome evaluation using standardized questionnaires. | 10 | 3 | 10 | 7.8-10 | 26  (90%) | 3  (10%) |
| 66 | Clinical Care | Follow-Up Assessment | Appropriate standardized outcome measures include Visual Analog Scale (VAS), Numeric Rating Scale (NRS), Knee injury osteoarthritis outcome score (KOOS) and Western Ontario and McMaster Universities Osteoarthritis Index (WOMAC) | 10 | 4 | 10 | 9-10 | 27  (93%) | 2  (7%) |
| 67 | Clinical Care | Follow-Up Assessment | Standardized questionnaires should be administered prior to the intervention and at 4–6 weeks, as well as 3, 6, 12, and 24 months after GAE. | 9 | 4 | 10 | 7-10 | 23  (79%) | 6  (21%) |
| 68 | Clinical Care | Follow-Up Assessment | Clinical success should be defined as achieving the minimum clinically important difference (MCID) (WOMAC: ≥ 12–15 points in total score; KOOS: ≥ 8–10 points in subscales; NRS/VAS: ≥ 2 points). | 9 | 3 | 10 | 7-10 | 24  (83%) | 5  (17%) |
| 69 | Clinical Care | Follow-Up Assessment | In addition to achieving predefined threshold improvements in outcome scores, subjective patient satisfaction with the treatment outcome should also be considered as clinical success in the context of patient-centered care.(Patient Global Assessment score of ≥7 on a 10-point scale) | 10 | 6 | 10 | 8-10 | 28  (97%) | 1  (3%) |
| 70 | Clinical Care | Repeat/Bilateral GAE | In patients with bilateral knee pain / bilateral OA with clear side predominance, the more painful knee should be treated first, and treatment of the contralateral side should be evaluated after at least 3 months. | 9 | 3 | 10 | 7-10 | 23  (79%) | 6  (21%) |
| 71  (1) | Clinical Care | Repeat/Bilateral GAE | In the absence of side predominance, both knees may be treated in a single session to avoid overloading the treated side after unilateral intervention. Patient-specific factors such as periprocedural anxiety or difficult vascular access may also constitute an indication for single-session bilateral GAE.  (p=1) | 8 | 0 | 10 | 5-9 | 19  (66%) | 10  (34%) |
| 71  (2) |  |  |  | 8 | 0 | 10 | 5-9 | 19  (66%) | 10  (34%) |
| 72 | Clinical Care | Repeat/Bilateral GAE | Before considering alternative treatment options, repeat GAE should be considered, and this possibility should be discussed with the patient prior to the initial treatment. | 8 | 3 | 10 | 7-9.3 | 23  (79%) | 6  (21%) |
| 73 | Clinical Care | Repeat/Bilateral GAE | Indications for repeat GAE include lack of pain reduction, defined as failure to achieve the MCID, or recurrence of pain, defined as initial achievement of the MCID followed by subsequent deterioration and loss of the MCID. | 8 | 0 | 10 | 7-10 | 22  (76%) | 7  (24%) |
| 74 | Clinical Care | Repeat/Bilateral GAE | Repeat GAE should be considered no earlier than 3 months after the initial procedure | 10 | 2 | 10 | 8-10 | 28  (97%) | 1  (3%) |
| 75 | Clinical Care | Repeat/Bilateral GAE | In repeat GAE, a change of the embolic agent (e.g., from temporary to permanent embolic agents) may be considered. | 9 | 5 | 10 | 8-10 | 25  (86%) | 4  (14%) |
